# Trends and Race/Ethnic Disparities in Diabetes-Related Hospital Use in Medicaid Enrollees: Analyses of Serial Cross-sectional State Data, 2008–2017

**DOI:** 10.1007/s11606-022-07842-5

**Published:** 2022-11-16

**Authors:** Puneet Kaur Chehal, Tegveer S. Uppal, Boon Peng Ng, Maria Alva, Mohammed K. Ali

**Affiliations:** 1 Department of Health Policy and Management, Rollins School of Public Health, Emory University, 1518 Clifton Road, Atlanta, GA 30322 USA; 2 Hubert Department of Global Health, Rollins School of Public Health, Emory University, Atlanta, GA USA; 3 College of Nursing, University of Central Florida, Orlando, FL USA; 4 Disability, Aging and Technology Cluster, University of Central Florida, Orlando, FL USA; 5 Massive Data Institute, McCourt School of Public Policy, Georgetown University, Washington, DC USA; 6 Department of Family and Preventive Medicine, School of Medicine, Emory University, Atlanta, GA USA

**Keywords:** Medicaid, disparities, diabetes, hospital use

## Abstract

**Background:**

Race/ethnic disparities in preventable diabetes-specific hospital care may exist among adults with diabetes who have Medicaid coverage.

**Objective:**

To examine race/ethnic disparities in utilization of preventable hospital care by adult Medicaid enrollees with diabetes across nine states over time.

**Design:**

Using serial cross-sectional state discharge records for emergency department (ED) visits and inpatient (IP) hospitalizations from the Healthcare Cost and Utilization Project, we quantified race/ethnicity-specific, state-year preventable diabetes-specific hospital utilization.

**Participants:**

Non-Hispanic Black, non-Hispanic White, and Hispanic adult Medicaid enrollees aged 18–64 with a diabetes diagnosis (excluding gestational or secondary diabetes) who were discharged from hospital care in Arizona, Iowa, Kentucky, Florida, Maryland, New Jersey, New York, North Carolina, and Utah for the years 2008, 2011, 2014, and 2017.

**Main Measures:**

Non-Hispanic Black-over-White and Hispanic-over-White rate ratios constructed using age- standardized state-year, race/ethnicity-specific ED, and IP diabetes-specific utilization rates.

**Key Results:**

The ratio of Black-over-White ED utilization rates for preventable diabetes-specific hospital care increased across the 9 states in our sample from 1.4 (CI 95, 1.31–1.50) in 2008 to 1.73 (CI 95, 1.68–1.78) in 2017. The cross-year-state average non-Hispanic Black-over-White IP rate ratio was 1.46 (CI 95, 1.42–1.50), reflecting increases in some states and decreases in others. The across-state-year average Hispanic-over-White rate ratio for ED utilization was 0.67 (CI 95, 0.63–0.71). The across-state-year average Hispanic-over-White IP hospitalization rate ratio was 0.72 (CI 95, 0.69–0.75).

**Conclusions:**

Hospital utilization by non-Hispanic Black Medicaid enrollees with diabetes was consistently greater and often increased relative to utilization by White enrollees within state programs between 2008 and 2017. Hispanic enrollee hospital utilization was either lower or indistinguishable relative to White enrollee hospital utilization in most states, but Hispanic utilization increased faster than White utilization in some states. Among broader patterns, there is heterogeneity in the magnitude of race/ethnic disparities in hospital utilization trends across states.

**Supplementary Information:**

The online version contains supplementary material available at 10.1007/s11606-022-07842-5.

## Introduction

Medicaid is a public health insurance program for low-income populations in the USA. Diabetes is a chronic metabolic condition that can result in major complications such as blindness, infections, vascular diseases, and limb amputations without effective disease management. Historically, diabetes has imposed substantial burden on Medicaid enrollees and state programs.^1–3^ There is growing recognition for the broader array of factors that can increase disease burden, and that may differentially affect racially/ethnically diverse subpopulations with the same Medicaid coverage.^4–8^ For instance, social determinants of health (SDH), e.g., food insecurity, can worsen health, and hinder realization of the benefits provided by Medicaid coverage (e.g., adhering to prescribed dietary guidelines) in some populations more than others. Surveillance of race/ethnic disparities in diabetes management could identify opportunities for reform.

There is broader research on race/ethnic disparities in diabetes disease management,^1, 9–15^ and some research on race/ethnic disparities in Medicaid programs.^16–18^ There is little work specifically evaluating racial/ethnic disparities among Medicaid enrollees with diabetes,^19, 20^ including disparities in hospital utilization. The need for emergency department (ED) and inpatient (IP) hospital care for diabetes complications are downstream indicators of poor access to ambulatory care. Despite Medicaid coverage, some patient populations may disproportionately experience other barriers in access to primary care providers.^21^ The ED may ultimately serve as the primary source of care for these patients.^22^ For others, the benefits of ambulatory care may be offset by other factors like mistrust or discrimination resulting in persistent complications.

Moreover, the experience of race/ethnic minority enrollees from any single state is not necessarily representative of disparities in other states because of heterogeneity in state populations and Medicaid program design. State-specific reforms to improve chronic disease care in Medicaid programs could also affect disparities over time.^23–25^ This study fills this gap in the research by exploring state-level race/ethnic disparities in potentially preventable ED and IP utilization for adult Medicaid enrollees in nine diverse states over 2008–2017.

## Methods

### Data Source and Population

We constructed state-level ED and IP utilization rates by race/ethnicity for Medicaid populations with diabetes in Arizona (AZ), Iowa (IA), Kentucky (KY), Florida (FL), Maryland (MD), New Jersey (NJ), New York (NY), North Carolina (NC), and Utah (UT).

We used ED discharge records from the State Emergency Department Databases (SEDD), and IP discharge records from the State Inpatient Databases (SID) produced by the Healthcare Cost and Utilization Project (HCUP) to measure hospital utilization.^26^ These databases account for *all* discharges at all hospitals in each state and include information on patient age, sex, and source of insurance. This study was Institutional Review Board exempt (Appendix I). For budgetary reasons, we selected a combination of state data with available race/ethnicity information, and that had diverse populations and Medicaid programs for 2008, 2011, 2014, and 2017. We used 2016 data for NY because 2017 data were not available.

We limited our analysis to records for non-elderly adults with Medicaid insurance ages 18–64 with a diabetes diagnosis (excluding gestational or secondary diabetes) indicated with International Classification of Diseases (ICD) codes (ICD-9 [250.XX] or ICD-10 [E10.XXX, E11.XXX, E13.XXX]).^27^ Individual SEDD and SID year datasets with greater than 1% missing demographic information were imputed using the Mice package in R version 4.1.2.^28^ Records for ED visits were restricted to visits that did not result in admission to avoid double counting discharges.

We classified race/ethnicity as non-Hispanic White, non-Hispanic Black, and Hispanic — White, Black, and Hispanic henceforth. Small sample issues precluded analysis of other race/ethnic groups. Race/ethnicity data were unavailable for IA, FL, and NC before 2011 and AZ before 2014. Data for these states in the specified years were excluded from our analysis. We also excluded state-year race data with ten or fewer discharges or that have relative standard errors of 30% or higher: KY Hispanic (ED and IP 2008–2011), UT Hispanic and Black (ED and IP 2008–2014), and MD Hispanic (ED 2008).

### Data Analysis

Our primary analysis focused on hospital utilization for potentially preventable diabetes-specific causes among Medicaid enrollees with diabetes diagnoses, which were identified using the Agency for Healthcare Research and Quality’s Prevention Quality Indicators (AHRQ’s PQIs).^29^ AHRQ’s PQIs include diagnostic codes for short-term diabetes complications, long-term diabetes complications, uncontrolled diabetes, lower extremity ulcers, inflammation, and infections for patients with diabetes (Appendix II Table 1). We augmented the AHRQ’s PQI conditions with additional diagnostic or procedure codes reflecting lower extremity amputation.^30, 31^ For sensitivity analysis, we also constructed state and race/ethnicity-specific trends in ED and IP use for adult Medicaid enrollees with diabetes *for all-causes*.

We constructed and analyzed state-year, race-specific utilization rates to account for different size populations within and across states. Specifically, we scaled total state-year ED or IP hospital discharges for Medicaid-insured, non-elderly, adult enrollees with diabetes by race/ethnicity group by the respective state-year count of non-elderly adult Medicaid enrollees in the race/ethnic group. Enrollee totals were derived using data from the American Community Survey (ACS) accessed via the IPUMS USA Database,^32^ which is designed to estimate annual representative demographic subpopulation counts for individual states.^33^ ACS population estimates were weighted using the *Survey* package in R. We standardized population estimates by age using the US adult population in 2010 from the Centers for Disease Control and Prevention (CDC) National Center for Health Statistics Wonder Database^34^ to account for differences in population age within and across states. Variance estimation for ED and IP hospitalization counts assumed a Poisson distribution; additional information is available in Appendix II.^35, 36^

We report ED visits and IP hospitalizations per 10,000 enrollees. To quantify disparities within states, we constructed Black-over-White and Hispanic-over-White rate ratios. 95% CI were used to evaluate the statistical significance of rate ratios and changes in underlying utilization rates over time.^35, 36^ Descriptive statistics separately show state total ED and IP discharge counts by subgroups.

## Results

State total ED or IP discharges for adult Medicaid enrollees with diabetes varied (Table 1). Potentially preventable diabetes-specific ED discharges generally accounted for about 6% of state total discharges, except for IA, which was at 10% of total ED discharges. The proportion of ED discharges among women varied between 62.1% in NC and 46.9% in NY. The majority of discharges in smaller states in our sample (IA, KY, and UT) were to White Medicaid-insured adults. MD had the largest proportion of Black ED discharges at 59.8%, and AZ had the largest proportion of Hispanic ED discharges at 36.6%. Diabetes-specific discharges accounted for 13–16% of state total IP discharges for Medicaid-insured adults with diabetes. Utilization across demographic groups was similar between ED and IP discharges.

**Table 1:** State Total Discharges for Non-elderly Adults with Diabetes Diagnosis and Medicaid Coverage, 2008, 2011, 2014, and 2017

	AZ	FL	IA	KY	MD	NC	NJ	NY^b^	UT
ED visits									
All-cause visits^a^	181,541	296,695	40,557	149,559	124,554	193,248	92,343	352,936	21,450
se	426.1	544.7	201.4	386.7	352.9	439.6	303.9	594.1	146.5
Diabetes-specific visits	11,589	17,953	4,033	10,008	8,494	11,766	6,309	26,833	1,395
se	107.7	134	63.5	100	92.2	108.5	79.4	163.8	37.3
Age, No. (%)^c^									
18–29	1,883	2,882	826	1,721	1,398	2,226	822	3,797	274
	(16.2)	(16.1)	(20.5)	(17.2)	(16.5)	(18.9)	(13.0)	(14.2)	(19.6)
30–44	4,012	5,629	1,379	3,412	2,637	3,928	1,754	7,420	534
	(34.6)	(31.4)	(34.2)	(34.1)	(31.0)	(33.4)	(27.8)	(27.7)	(38.3)
45–64	5,694	9,442	1,828	4,875	4,459	5,612	3,733	15,616	587
	(49.1)	(52.6)	(45.3)	(48.7)	(52.5)	(47.7)	(59.2)	(58.2)	(42.1)
Female, No. (%)^c^	5,525	10,491	2,064	5,528	4,306	7,306	3,018	12,581	806
	(47.7)	(58.4)	(51.2)	(55.2)	(50.7)	(62.1)	(47.8)	(46.9)	(57.8)
Race/ethnicity, No. (%)^c^									
Black	1,090	6,814	587	1,607	5,076	5,667	2,789	10,143	51
	(9.4)	(38.0)	(14.6)	(16.1)	(59.8)	(48.2)	(44.2)	(37.8)	(3.7)
Hispanic	4,243	3,359	160	101	246	296	1,276	5,497	221
	(36.6)	(18.7)	(4.0)	(1.0)	(2.9)	(2.5)	(20.2)	(20.5)	(15.8)
White	4,986	7,303	3,024	8,130	2,555	4,641	1,776	7,278	915
	(43.0)	(40.7)	(75.0)	(81.2)	(30.1)	(39.4)	(28.2)	(27.1)	(65.6)
Other	1,270	477	262	170	617	1,162	468	3,915	208
	(11.0)	(2.7)	(6.5)	(1.7)	(7.3)	(9.9)	(7.4)	(14.6)	(14.9)
IP hospitalizations									
All-cause visits^a^	89,702	202,290	22,473	77,126	72,064	90,322	58,211	320,890	9,212
se	299.5	449.8	149.9	277.7	268.4	300.5	241.3	566.5	96
Diabetes-specific visits	14,734	29,377	3,759	10,471	10,692	15,064	8,342	44,589	1,559
se	121.4	171.4	61.3	102.3	103.4	122.7	91.3	211.2	39.5
Age, No. (%)^c^									
18–29	3,270	5,658	971	2,255	2,173	3,740	1,419	7,390	436
	(22.2)	(19.3)	(25.8)	(21.5)	(20.3)	(24.8)	(17.0)	(16.6)	(28.0)
30–44	4,666	7,996	1,169	3,254	2,981	4,707	2,000	11,197	491
	(31.7)	(27.2)	(31.1)	(31.1)	(27.9)	(31.2)	(24.0)	(25.1)	(31.5)
45–64	6,798	15,723	1,619	4,962	5,538	6,617	4,923	26,002	632
	(46.1)	(53.5)	(43.1)	(47.4)	(51.8)	(43.9)	(59.0)	(58.3)	(40.5)
Female, No. (%)^c^	6,291	15,371	1,786	5,471	4,996	8,459	3,824	19,208	882
	(42.7)	(52.3)	(47.5)	(52.2)	(46.7)	(56.2)	(45.8)	(43.1)	(56.6)
Race/ethnicity, No. (%)^c^									
Black	1,121	10,051	446	1,398	6,042	6,624	3,388	16,066	46
	(7.6)	(34.2)	(11.9)	(13.4)	(56.5)	(44.0)	(40.6)	(36.0)	(3.0)
Hispanic	4,570	5,403	122	113	398	341	1,620	10,090	244
	(31.0)	(18.4)	(3.2)	(1.1)	(3.7)	(2.3)	(19.4)	(22.6)	(15.7)
White	6,925	13,136	2,886	8,765	3,554	6,323	2,796	11,727	848
	(47.0)	(44.7)	(76.8)	(83.7)	(33.2)	(42.0)	(33.5)	(26.3)	(54.4)
Other	2,118	787	305	195	698	1,776	538	6,706	421
	(14.4)	(2.7)	(8.1)	(1.9)	(6.5)	(11.8)	(6.4)	(15.0)	(27.0)
ACA Medicaid Exp.^b^	Yes	-	Yes	Yes	Yes	-	Yes	Yes	-

^a^Counts of ED visits and IP stays reflect sums of all stays from years 2008, 2011, 2014, and 2017 (or 2016 if NY) from HCUP State Emergency Department Database and State Inpatient Databases

^b^Characteristics for year 2016 provided for New York due to HCUP data availability at time of analysis

^c^Percent in parentheses reflects proportion of total discharges

^d^ACA Medicaid Exp. stands for Affordable Care Act Medicaid Expansion, where Yes implies the state implemented the expansion in 2014

**Fig. 1:**
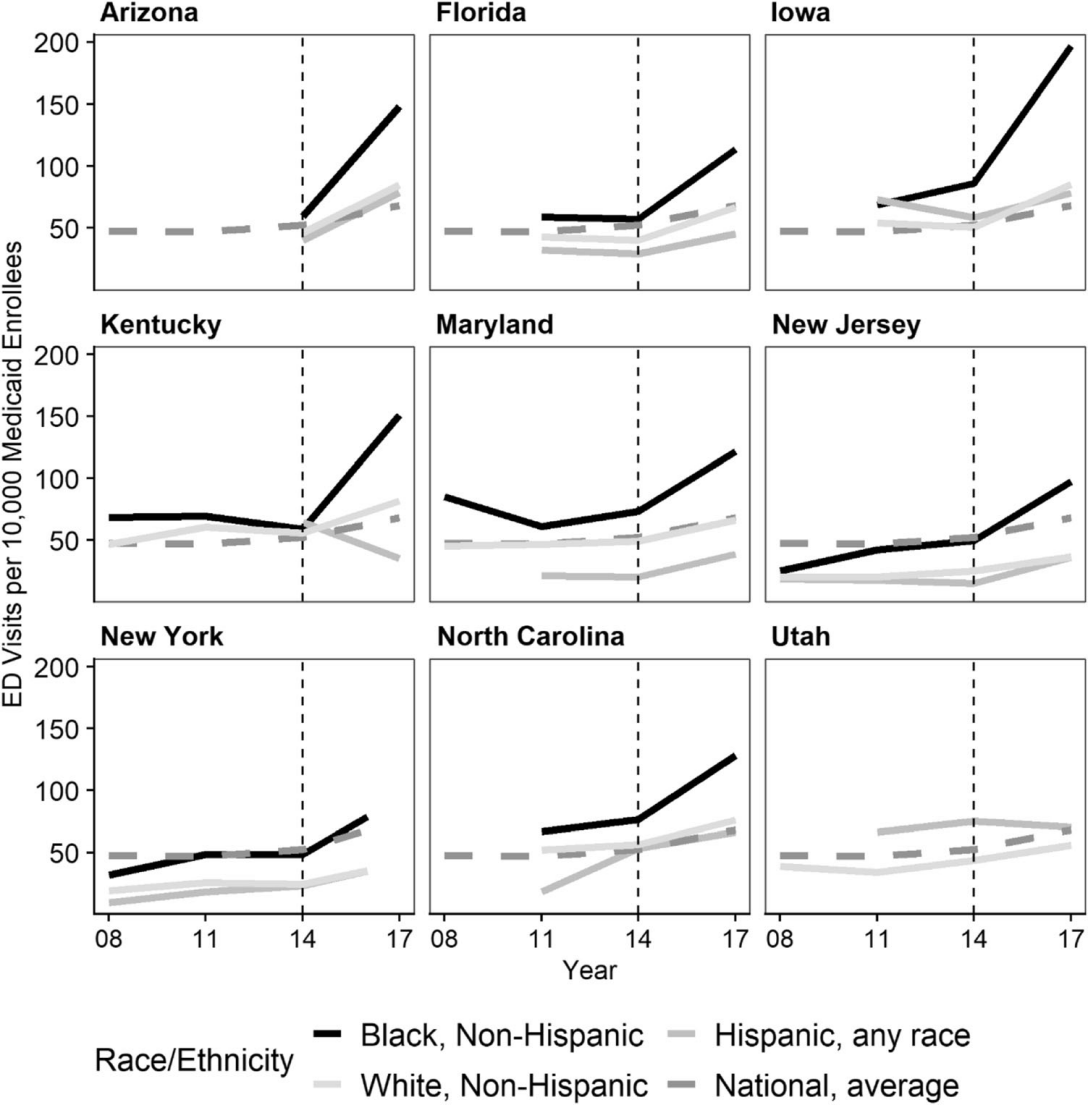
State diabetes-specific ED visits for adult Medicaid enrollees with diabetes by race/ethnicity. Source/notes: ED utilization rates constructed using ED visit count data from the HCUP State Emergency Department Databases and population data from the IPUMS USA American Community Survey for 2008, 2011, 2014, and 2017 (NY 2016 data). All rates were age-standardized to the 2010 US adult population using population estimates available from the CDC Wonder Database. Only Medicaid-insured non-elderly adults with a diabetes diagnosis are included in the sample. NEDS data was used to estimate national diabetes-related ED visits for all non-elderly adult Medicaid enrollees. Dashed line indicates full implementation of the ACA

**Fig. 2:**
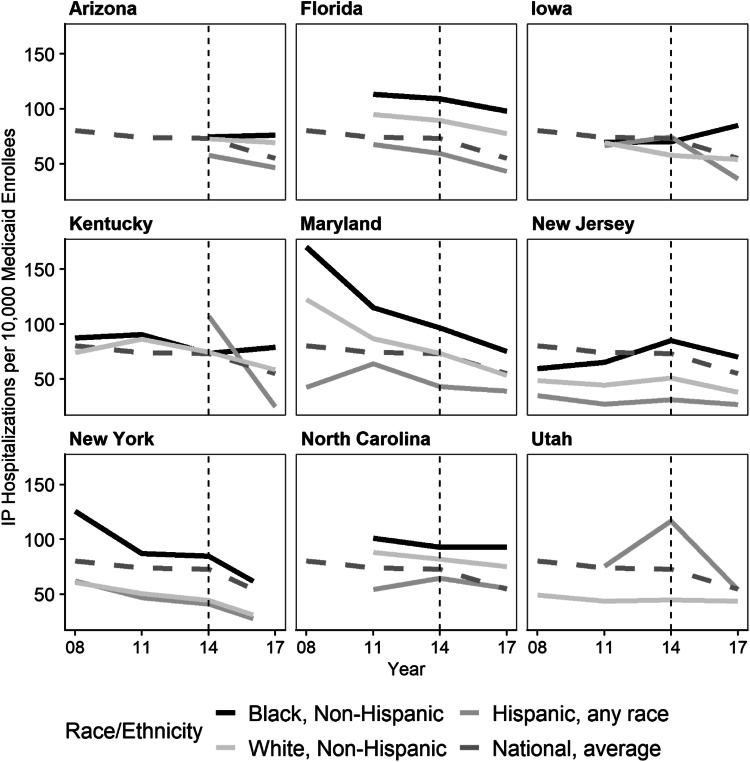
State diabetes-specific IP hospitalizations for adult Medicaid enrollees with diabetes by race/ethnicity. Source/notes: IP utilization rates by state sourcing numerator data from the HCUP State Inpatient Databases and denominator data from the IPUMS USA American Community Survey from years 2008 to 2017 (NY 2016). All rates were age-standardized to the 2010 US adult population using population estimates available from the CDC Wonder Database. Only Medicaid-insured non-elderly with a diabetes diagnosis are included in the sample. NISS data was used to estimate national diabetes-related IP hospitalizations for all non-elderly adult Medicaid enrollees. Dashed line indicates full implementation of the ACA

Consistent with broader trends in hospital utilization for all adults with diabetes between 2008 and 2017,^37, 38^ we observed almost universal increases in ED visits (Fig. 1 and Appendix II Table 2) and generally downward trends in IP hospitalizations (Fig. 2 and Appendix II Table 3).

### Hospital Utilization for Preventable Diabetes-Specific ED Visits

Black enrollees had higher ED utilization rates for preventable diabetes conditions across years and states relative to White enrollees amounting to an average Black-over-White rate ratio of 1.54 (CI 95, 1.48–1.60) (Table 2). NJ had the highest cross-year average rate ratio of 2.09 (CI 95, 2.03–2.15). KY had the lowest average rate ratio at 1.42 (CI 95, 1.40–1.44). Black-over-White rate ratios increased across states (increasing disparity) from an average of 1.4 (CI 95, 1.31–1.5) in 2008 to 1.73 (CI 95, 1.68–1.78) in 2017. Between 2008 and 2017, the underlying utilization rates for Black enrollees increased from an average of 47.7 to 126.8 visits per 10,000 enrollees, whereas, for White enrollees, rates increased from 34.1 to 73.5 visits per 10,000 enrollees (Fig. 1 and Appendix II Table 2). For comparison, we also reported survey-weighted nationally representative ED and IP utilization (Fig. 1) for all non-elderly adult Medicaid enrollees using data from the Nationwide Emergency Department Sample (NEDS) and the National Inpatient Sample (NIS).^26^ Nationally, the ED visit rate remained around 50 visits per 10,000 enrollees between 2008 and 2014 after which it began a slow upward trend.

**Table 2:** ED Visits Race/Ethnicity-Specific Rate Ratios for Adult Medicaid Enrollees with Diabetes^a^

Black to White rate ratio (CI 95%)	2008^b^	2011^b^	2014^b^	2017^c^	Average, 2008–2017
AZ	-	-	1.29	1.75	1.59
			(1.21, 1.38)	(1.69, 1.81)	(1.55, 1.63)
FL	-	1.38	1.42	1.71	1.54
		(1.30, 1.47)	(1.33, 1.51)	(1.65, 1.77)	(1.51, 1.57)
IA	-	1.27	1.70	2.31	1.85
		(1.19, 1.36)	(1.60, 1.81)	(2.23, 2.39)	(1.82, 1.88)
KY	1.47	1.14	1.06	1.85	1.42
	(1.38, 1.57)	(1.08, 1.21)	(1.00, 1.12)	(1.79, 1.91)	(1.40, 1.44)
MD	1.88	1.31	1.50	1.83	1.65
	(1.76, 2.00)	(1.23, 1.40)	(1.42, 1.59)	(1.76, 1.91)	(1.62, 1.68)
NC	-	1.30	1.35	1.69	1.47
		(1.23, 1.37)	(1.28, 1.42)	(1.63, 1.75)	(1.44, 1.50)
NJ	1.23	2.07	1.98	2.66	2.09
	(1.07, 1.41)	(1.84, 2.33)	(1.80, 2.18)	(2.50, 2.83)	(2.03, 2.15)
NY^a^	1.66	1.89	1.98	2.23	1.98
	(1.49, 1.86)	(1.74, 2.05)	(1.83, 2.15)	(2.11, 2.36)	(1.94, 2.03)
UT	-	-	-	1.89	1.89
				(1.78, 2.01)	(1.78, 2.01)
Average^d^	1.40	1.42	1.48	1.73	1.54
	(1.31, 1.50)	(1.36, 1.49)	(1.42, 1.55)	(1.68, 1.78)	(1.48, 1.60)
Hispanic to White rate ratio (CI 95%)	2008^b^	2011^b^	2014	2017^c^	Average, 2008–2017
AZ	-	-	0.87	0.93	0.91
			(0.81, 0.93)	(0.89, 0.97)	(0.89, 0.94)
FL	-	0.75	0.73	0.68	0.71
		(0.69, 0.81)	(0.67, 0.79)	(0.64, 0.72)	(0.69, 0.73)
IA	-	1.36	1.16	0.92	1.11
		(1.26, 1.46)	(1.07, 1.25)	(0.87, 0.97)	(1.08, 1.14)
KY	-	-	1.16	0.43	0.46
			(1.09, 1.24)	(0.39, 0.47)	(0.44, 0.48)
MD	-	0.45	0.41	0.58	0.42
		(0.39, 0.52)	(0.36, 0.47)	(0.54, 0.63)	(0.40, 0.44)
NC	-	0.35	0.94	0.88	0.75
		(0.30, 0.41)	(0.87, 1.01)	(0.83, 0.93)	(0.73, 0.77)
NJ	0.93	0.88	0.61	0.99	0.86
	(0.79, 1.09)	(0.75, 1.03)	(0.52, 0.71)	(0.91, 1.07)	(0.83, 0.89)
NY^a^	0.51	0.71	0.94	0.98	0.82
	(0.42, 0.62)	(0.63, 0.80)	(0.85, 1.04)	(0.91, 1.05)	(0.79, 0.85)
UT	-	1.95	1.72	1.26	1.52
				(1.18, 1.35)	(1.48, 1.56)
Average^d^	0.40	0.57	0.69	0.74	0.67
	(0.35, 0.46)	(0.53, 0.61)	(0.65, 0.73)	(0.71, 0.77)	(0.63, 0.71)

^a^Rate ratios calculated using ED utilization rates constructed using ED visit count data from the HCUP State Emergency Department Databases and population data from the IPUMS USA American Community Survey for 2008, 2011, 2014, and 2017. All rates were age-standardized to the 2010 US adult population using population estimates available from the CDC Wonder Database

^b^indicates estimate was suppressed. All estimates *n*≤10 events or RSE≥30% were not reported. Additionally, HCUP prioritizes ethnicity when coding the uniform race variable, FL, IA, and NC did not provide an ethnicity variable in 2008. AZ did not provide an ethnicity variable in 2008 and 2011

^c^Rate ratios for NY were calculated from ED utilization rates from 2016 as 2017 data was not available for NY

^d^Average rate ratios for ED visits constructed using pooled data from states AZ, FL, IA, KY, MD, NC, NJ, NY, and UT. Denominator population estimates generated from pooled state populations estimated from the IPUMS USA American Community Survey for corresponding years

The across-state-year average Hispanic-over-White rate ratio for ED utilization was 0.67 (CI 95, 0.63–0.71) (Table 2). In most states, utilization rates for Hispanic Medicaid enrollees remained either lower than or indistinguishable from White enrollee rates. The exceptions were UT which had an average Hispanic-over-White rate ratio of 1.52 (CI 95, 1.48–1.56) and IA in 2011 with a ratio of 1.11 (CI 95, 1.08–1.14). MD had the lowest rate ratio of 0.42 (CI 95, 0.40–0.44). The Hispanic-over-White rate ratios significantly increased in magnitude in AZ, MD, and NY, and either increased inconsistently (NC and NJ) or decreased in other states (IA, KY, and UT). Temporal or consistent increases in state rate ratios reflected larger increases in underlying Hispanic ED utilization rates relative to White rates from the same state. For instance, NY’s rate ratio increased between 2008 and 2017 because the ED rate for Hispanic enrollees grew relatively more, from 9.7 (CI 95, 8.8–10.6) to 34.7 (CI 95, 33.3–36.0) visits per 10,000 enrollees than the rate for White enrollees, from 19.1 (CI 95, 18.8–20.4) to 35.3 (CI 95, 34.0–36.5) visits per 10,000 enrollees (Fig. 1 and Appendix II Table 2).

The top five primary diagnostic codes for diabetes-specific ED visits were generally for acute or short-term complications (Appendix II Table 4). In 2008, the most frequent reason for ED visits across all race/ethnic groups was a broad code for diabetes with any related complications. The second most common diagnostic codes were for uncontrolled diabetes in Black and Hispanic adults and lower limb cellulitis in White patients. In 2017, the most common reason for ED visits was hyperglycemia among type 2 diabetes patients in all race/ethnic groups, followed by hypoglycemia in Black and Hispanic adults and type 1-related hyperglycemia in White patients.

### Hospital Utilization for Preventable Diabetes-Specific IP Hospitalizations

The cross-year-state average Black-over-White IP rate ratio was 1.46 (CI 95, 1.42–1.5) (Table 3), reflecting generally higher Black enrollee diabetes-specific preventable hospitalization rates compared to White enrollees across states over time. Among states with data for 2008 onwards, the highest and lowest average rate ratios were in NY at 1.91 (CI 95, 1.88–1.94) and KY at 1.13 (CI 95, 1.11–1.15), respectively. Over time, rate ratios increased in NC, IA, and NJ; varied in KY and NY; and experienced little or no significant change in AZ, FL, and MD. State rate ratios changed in the same direction but for different reasons. For instance, Black-over-White rate ratios for IA and NJ increased over time. In IA, White hospitalizations decreased while Black hospitalizations increased, whereas, in NJ, there were episodic increases in hospitalization for Black enrollees but no equivalent increases for White enrollees (Fig. 2 and Appendix II Table 3). Nationally, the IP visit rate for Medicaid enrollees with diabetes trended down between 2008 and 2017.

**Table 3:** IP Hospitalizations Race/Ethnicity-Specific Rate Ratios for Adult Medicaid Enrollees with Diabetes^a^

Black to White rate ratio (CI 95%)	2008^b^	2011^b^	2014^b^	2017^c^	Average, 2008–2017
AZ	-	-	1.02	1.10	1.06
			(0.97, 1.07)	(1.05, 1.16)	(1.03, 1.09)
FL	-	1.20	1.22	1.26	1.22
		(1.16, 1.24)	(1.18, 1.26)	(1.22, 1.31)	(1.20, 1.24)
IA	-	1.00	1.21	1.58	1.24
		(0.94, 1.06)	(1.14, 1.29)	(1.49, 1.67)	(1.21, 1.27)
KY	1.18	1.05	0.99	1.35	1.13
	(1.13, 1.24)	(1.01, 1.10)	(0.94, 1.04)	(1.29, 1.42)	(1.11, 1.15)
MD	1.39	1.32	1.31	1.41	1.36
	(1.35, 1.43)	(1.27, 1.37)	(1.26, 1.37)	(1.34, 1.49)	(1.34, 1.38)
NC	-	1.15	1.13	1.24	1.17
		(1.11, 1.19)	(1.09, 1.17)	(1.19, 1.29)	(1.15, 1.19)
NJ	1.23	1.47	1.67	1.85	1.54
	(1.15, 1.31)	(1.38, 1.57)	(1.58, 1.76)	(1.73, 1.97)	(1.51, 1.57)
NY^a^	2.06	1.72	1.89	1.97	1.91
	(1.98, 2.14)	(1.64, 1.80)	(1.80, 1.98)	(1.85, 2.10)	(1.88, 1.94)
UT	-	-	-	1.96	1.96
				(1.82, 2.11)	(1.82, 2.11)
Average^d^	1.78	1.36	1.40	1.42	1.46
	(1.73, 1.83)	(1.32, 1.40)	(1.36, 1.44)	(1.37, 1.47)	(1.42, 1.50)
Hispanic to White rate ratio (CI 95%)	2008^b^	2011^b^	2014	2017^c^	Average, 2008–2017
AZ	-	-	0.79	0.67	0.73
			(0.75, 0.83)	(0.63, 0.71)	(0.71, 0.75)
FL	-	0.71	0.66	0.56	0.65
		(0.68, 0.74)	(0.63, 0.69)	(0.53, 0.59)	(0.64, 0.66)
IA	-	0.96	1.29	0.67	0.98
		(0.89, 1.03)	(1.21, 1.38)	(0.61, 0.74)	(0.95, 1.01)
KY	-	-	1.44	0.42	0.58
			(1.38, 1.51)	(0.37, 0.47)	(0.57, 0.60)
MD	0.35	0.74	0.59	0.73	0.56
	(0.32, 0.38)	(0.70, 0.79)	(0.55, 0.63)	(0.67, 0.79)	(0.55, 0.57)
NC	-	0.62	0.79	0.74	0.71
		(0.58, 0.66)	(0.75, 0.84)	(0.70, 0.79)	(0.69, 0.73)
NJ	0.72	0.72	0.61	0.71	0.66
	(0.66, 0.78)	(0.66, 0.78)	(0.56, 0.66)	(0.65, 0.78)	(0.64, 0.68)
NY^a^	1.02	0.92	0.92	0.89	0.95
	(0.97, 1.07)	(0.87, 0.97)	(0.87, 0.98)	(0.82, 0.97)	(0.93, 0.97)
UT	-	1.72	2.60	1.23	1.87
		(1.60, 1.85)	(2.43, 2.78)	(1.13, 1.34)	(1.83, 1.91)
Average^d^	0.88	0.71	0.73	0.64	0.72
	(0.85, 0.92)	(0.68, 0.74)	(0.70, 0.76)	(0.61, 0.67)	(0.69, 0.75)

^a^Rate ratios calculated using IP utilization rates by state sourcing numerator data from the HCUP State Inpatient Databases and denominator data from the IPUMS USA American Community Survey from years 2008 to 2017. All rates were age-standardized to the 2010 US adult population using population estimates available from the CDC Wonder Database

^b^indicates estimate was suppressed. All estimates *n*≤10 events or RSE≥30% were not reported. Additionally, HCUP prioritizes ethnicity when coding the uniform race variable, FL, IA, and NC did not provide an ethnicity variable in 2008. AZ did not provide an ethnicity variable in 2008 and 2011

^c^Rate ratios for NY were calculated from IP utilization rates from 2016 as 2017 data was not available for NY

^d^Average inpatient stays aggregated from pooled SID data from states AZ, FL, IA, KY, MD, NC, NJ, NY, and UT. Denominator population estimates generated from pooled state populations estimated from the IPUMS USA American Community Survey for corresponding years

The average Hispanic-over-White IP hospitalization rate ratio for preventable diabetes-specific conditions was 0.72 (CI 95, 0.69–0.75) (Table 3). IP rates for Hispanic enrollees were almost always lower than those for White enrollees in all states but IA and UT, which had the highest average rate ratios of 0.98 (CI 95, 0.95–1.01) and 1.87 (CI 95, 1.83–1.91), respectively. MD had the lowest rate ratio of 0.56 (CI 95, 0.55–0.57). Rate ratios significantly decreased consistently or episodically in AZ, FL, KY, and NY; increased in MD and NC; and fluctuated over time in MD, NC, and NY. In states that experienced decreasing rate ratios, underlying Hispanic IP rates decreased relatively more than White rates. For example, in FL, Hispanic IP rates decreased from 67.5 (CI 95, 64.0–71.1) in 2011 to 43.1 (CI 95, 40.8–45.4) visits per 10,000 in 2017; whereas, White IP rates decreased from 94.8 (CI 95, 91.5–98.0) to 77.6 (CI 95, 75.0–80.1) visits per 10,000. In states for which ratios fluctuated over time, underlying rates show that White enrollees’ IP rates trended downwards while Hispanic rates experienced larger changes in either direction.

The top five reasons for IP hospitalizations were comparable across race/ethnic groups (Appendix II Table 5). The most frequent diagnosis code in both 2008 and 2017 was diabetic ketoacidosis. Lower limb cellulitis was frequently in the top five reasons for admission among White and Hispanic patients, while uncontrolled diabetes was frequently coded for hospitalized Black patients.

### All-Cause Hospital Utilization

We find that race/ethnicity-specific utilization patterns for preventable diabetes-specific causes extend to *all* hospital utilization by adult Medicaid enrollees with diabetes (Appendix II Tables 6–9 and Figs. 1 and 2).

## Discussion

In our nine-state analysis of ED and IP hospital use for potentially preventable diabetes conditions among non-elderly adult Medicaid enrollees with diabetes, utilization trends were almost always greater for Black Medicaid enrollees than for White Medicaid enrollees. Black-White disparities in ED visits have grown in all states, and in some states, disparities in IP hospitalizations have also grown. Hispanic enrollee hospital utilization was either lower or indistinguishable relative to White enrollee hospital utilization in most states, but Hispanic utilization increased faster than White utilization in some states. Among broader patterns, we documented considerable heterogeneity in the magnitude of race/ethnic disparities in hospital utilization trends across states. The most common reasons for ED and IP hospital use were generally similar across race/ethnic groups but second most common reasons varied across groups.

This study extends the broader literature on Black-White disparities in diabetes prevalence, management, and complications^1, 9–11^ by showing persistent disparities in preventable utilization of hospital care for diabetes-specific complications *within* nine state Medicaid programs. Multifactorial system-, provider-, and individual-level factors that cause disparities in access to quality healthcare and health outcomes^39^ can persist among racially/ethnically diverse Medicaid-insured enrollee populations. As such, race/ethnicity in our analysis is likely a proxy for broader societal barriers that lead to higher hospital use among Black enrollees. Specifically, our evidence of disparities in preventable diabetes-specific hospital utilization in Medicaid programs could result from pre-existing disparities in the availability of providers and the quality/comprehensiveness of care offered to segregated low-income communities.^39, 40^ Disparities in SDH among Medicaid enrollee populations can also undermine access to and effectiveness of care, e.g., food security, open space for exercise, or transportation.^41, 42^ Disparities in access to quality care could also result from factors at the provider level (e.g., provider bias) or at the individual level (e.g., race/ethnicity-specific patient beliefs).^39^

Our evidence of Black-White disparities in potentially preventable diabetes-specific hospital utilization in Medicaid may reflect lower medication adherence — which has been previously observed in Black Medicaid enrollees with diabetes.^19^ Lower medication adherence makes Black enrollees more vulnerable to diabetes-related complications and comorbidities that would increase their need for hospital care.^43–45^ Studies have linked lack of trust in the healthcare systems with poorer self-management and medication adherence among Black patients with diabetes.^12, 13^ Black populations in the USA have reason to mistrust health system actors given historical context, e.g., the Tuskegee experiment or Henrietta Lacks. We observed that Black Medicaid enrollees using hospital care were more likely to be diagnosed with uncontrolled diabetes than White patients. However, our evidence of Black-White disparities in utilization of hospital care by Medicaid-insured adults with diabetes for *all* causes suggests systemic factors may explain observed disparities, not just diabetes-specific medication adherence. Lastly, provider stereotypical assumptions about minority patients or mismatched provider communication styles can also undermine adherence to prescribed therapies.^39^

We do not observe consistent Hispanic White disparities in IP and ED utilization despite previous research that documents worse quality of care, and lower medication adherence in Hispanic patients with diabetes relative to non-Hispanic White patients.^1, 14, 15^ Our findings are consistent with data from the National Hospital Ambulatory Medical Care Surveys, which shows that US Hispanic ED utilization overall is close to and sometimes lower than White people, and with findings from other research on hospital use.^46, 47^ The difference in Black and Hispanic trends is supported by the broader literature on health disparities which emphasizes the role of minority-specific factors.^48, 49^ This difference in Black and Hispanic disparities in hospital use may reflect underutilization of hospital care by Hispanic patients or other unique factors such as migration patterns.^50^

We document substantial differential changes in disparities across states. Even among increases in Black-White disparities in ED utilization common to all states, there was variation in the magnitude of increases between states. In addition to broader differences across states, state Medicaid programs can differ and evolve in ways that could influence disparities in quality care and health outcomes. Program design can directly affect race/ethnic disparities, e.g., by requiring provider cultural competency training, or recognition of SDH.^3, 40, 51^ Indirectly, program design can also affect race/ethnic disparities, e.g., by the type of provider reimbursement or benefit generosity.^3, 51^

There are some limitations to our study. We cannot explore whether diabetes-specific hospitalizations are undercounting undiagnosed adults or overcounting visits due to high repeated use by a small segment of adults with diabetes. Because there are no state-level annual race/ethnicity-specific data on the total number of Medicaid enrollees with diabetes, we cannot construct annual state hospital utilization rates specifically for adults with diabetes by race/ethnicity. Instead, our rates reflect both disparities in access to quality care and disparities in diabetes prevalence; therefore, race/ethnic groups with higher diabetes prevalence have higher hospital utilization in our analysis. The broader upward trends in ED visits and downward trends in IP hospitalizations shown in our data and earlier studies^37, 52^ could be in part due to the transition from ICD-9 to ICD-10 coding, as well as the ACA Medicaid expansion. Since the coding or policy changes are constant across subpopulations within states, the coding change should not affect our conclusions regarding disparities. Sample size limitations prevented rural/urban comparisons. Lastly, it is possible that evolving use of race/ethnic identifiers influenced our trend estimates by changing the composition of demographic subgroups.

Poorly managed diabetes increases risk of substantial diabetes-related complications and disability for patients.^1–3^ The existence of race/ethnic disparities in preventable utilization of hospital care among Medicaid enrollees with diabetes also contributes to the ever-increasing program expenditures associated with the increased prevalence of diabetes.^53^ As noted, some states are directly addressing race/ethnic disparities in their Medicaid programs, but the approaches vary and the comparative effects are largely unknown at this time.^51^ More policy and translation research is needed.^54, 55^

## Supplementary Information

ESM 1 (DOCX 730 kb)
