# Supplementary material for: Trends and Race/Ethnic Disparities in Diabetes-Related Hospital Use in Medicaid Enrollees: Analyses of Serial Cross-sectional State Data, 2008–2017
Source: J Gen Intern Med. 2022 Nov 16;38(10):2279–88. doi: 10.1007/s11606-022-07842-5 (PMC10406763; doi:10.1007/s11606-022-07842-5)
Supplement: Supplementary file 1 — (DOCX 730 kb) [file 11606_2022_7842_MOESM1_ESM.docx]

**Appendix I: IRB Documentation**

**Appendix II: Supplemental Materials**

**Table of Contents**

[**eTable 1 ICD-9/ICD-10 and PCS Codes Used to Identify Potentially Preventable Diabetes-Specific Hospital Events** 2](#_Toc104230736)

[**Appendix 1. Construction of Age-Standardized Rate Ratios** 4](#_Toc104230737)

[**eTable 2 Age-adjusted Rates of Diabetes Specific ED Visits per 10,000 Adult Medicaid Enrollees by Race/Ethnicity** 5](#_Toc104230738)

[**eTable 3 Age-adjusted Rates of Diabetes-Specific IP Hospitalizations Visits per 10,000 Adult Medicaid Enrollees by Race/Ethnicity** 6](#_Toc104230739)

[**eTable 4 Most Common Diagnostic Codes by Race/Ethnicity: ED** 7](#_Toc104230740)

[**eTable 6 Age-adjusted Rates of All-Cause ED Visits per 10,000 Adult Medicaid Enrollees by Race/Ethnicity** 12](#_Toc104230741)

[**eTable 7 Age-adjusted Rates of All-Cause IP Hospitalizations Visits per 10,000 Adult Medicaid Enrollees by Race/Ethnicity** 13](#_Toc104230742)

[**eFigure 1 State All-Cause ED Visits for Adult Medicaid Enrollees with Diabetes by Race/Ethnicity** 14](#_Toc104230743)

[**eFigure 2 State All-Cause IP Visits for Adult Medicaid Enrollees with Diabetes by Race/Ethnicity** 15](#_Toc104230744)

[**eTable 8 Rate Ratios of Average Annual Recorded Emergency Department Visits - All Cause** 16](#_Toc104230745)

[**eTable 9 Rate Ratios of Average Annual Recorded Inpatient Hospitalizations - All Cause** 17](#_Toc104230746)

# **eTable 1 ICD-9/ICD-10 and PCS Codes Used to Identify Potentially Preventable Diabetes-Specific Hospital Events**

|  | **ICD-9-CM/PCS** | **ICD-10-CM/PCS** |
| --- | --- | --- |
| **Short-Term Complications^1^** | 250.10, 250.11, 250.12, 250.13, 250.20, 250.21, 250.22, 250.23, 250.30, 250.31, 250.32, 250.33 | E10.10, E10.11, E10.65, E10.69,  E11.00, E11.01, E11.10, E11.641, E11.65, E11.69, E13.00, E13.01, E13.10, E13.641, E13.65, E13.69 |
| **Long-Term Complications^1^** | 250.4, 250.40, 250.41, 250.42, 250.43, 250.5, 250.51, 250.52, 250.53, 250.6, 250.61, 250.62, 250.63, 250.7, 250.70, 250.71, 250.72, 250.73, 250.8, 250.80, 250.81, 250.82, 250.83, 250.9, 250.90, 250.91, 250.92, 250.93 | E1021, E1029, E10311, E10319, E1036, E1039, E1040, E1051, E1052, E1059, E10618, E10620, E10621, E10622, E10628, E10630, E10638, E10649 E1065, E1069, E108, E118, E1121, E1129, E11311, E1136, E11319, E1139, E1140, E1151, E1152, E1159, E11618, E11620, E11621, E11622, E11628, E11630, E11638, E11649, E1165, E1169, E1321, E1322, E1329, E13311, E13319, E1336, E1339, E1340, E1351, E1352, E1359, E13618, E13620, E13621, E13622, E13628, E13630, E13638, E13649, E1365, E1369 |
| **Uncontrolled Diabetes^1^** | 250.02, 250.03 | E10649, E1065, E1165, E11649,  E13649, E1365 |
| **Lower Extremity Ulcers, Inflammation, and Infection^2^** | 454.0, 707.1, 680.6, 680.7, 681.1, 682.6, 682.7, 711.05, 711.06, 711.07, 730.05,730.06, 730.07, 730.15, 730.16, 730.17, 730.25, 730.26, 730.27, 730.35, 730.36, 730.37, 730.85, 730.86, 730.87, 730.95, 730.96, 730.97, 785.4 | L97, I83.009, I83.019, 183.029, L02.429, L02.439, L02.629, L02.639, L03.039, L03.049, L03.129, L03.119, M00.059, M00.159, M00.259, M00.859, M00.069, M00.169, M00.269, M00.869, M00.079, M00.179, M00.279, M00.879, M86.159, M86.259, M86.169, M86.269, M86.179, M86.279, M86.659, M86.669, M86.679, M86.9, M90.859, M90.869, M90.879, I96 |
| **Lower Extremity Amputations^1, 3^** | 8410, 8411, 8412, 8413, 8414, 8415, 8416, 8417, 8418, 8419  Excluded:  895.x, 896.x, 897.x | 0Y6C0Z1, 0Y6C0Z2, 0Y6C0Z3, 0Y6D0Z1, 0Y6D0Z2, 0Y6D0Z3, 0Y6H0Z1, 0Y6H0Z2, 0Y6H0Z3, 0Y6J0Z1, 0Y6J0Z2, 0Y6J0Z3, 0Y6P0Z0, 0Y6P0Z2, 0Y6P0Z3, 0Y6Q0Z0, 0Y6Q0Z1, 0Y6Q0Z2, 0Y6Q0Z3, 0Y6R0Z0, 0Y6R0Z1, 0Y6R0Z2, 0Y6R0Z3, 0Y6S0Z0, 0Y6S0Z1, 0Y6S0Z2, 0Y6S0Z3, 0Y6T0Z0, 0Y6T0Z1, 0Y6T0Z2, 0Y6T0Z3, 0Y6U0Z0, 0Y6U0Z1, 0Y6U0Z2, 0Y6U0Z3, 0Y6V0Z0, 0Y6V0Z1, 0Y6V0Z2, 0Y6V0Z3, 0Y6W0Z0, 0Y6W0Z1, 0Y6W0Z2, 0Y6W0Z3, 0Y6X0Z0, 0Y6X0Z1, 0Y6X0Z2, 0Y6X0Z3, 0Y6Y0Z0, 0Y6Y0Z1, 0Y6Y0Z2, 0Y6Y0Z3, 0Y6M0Z4", 0Y6M0Z5, 0Y6M0Z6, Y6M0Z7, 0Y6M0Z8, 0Y6M0Z9, 0Y6M0ZB, 0Y6M0ZC, 06YM0ZD, 06YM0ZF, 0Y6N0Z4, 0Y6N0Z5, 0Y6N0Z6, 0Y6N0Z7, 0Y6N0Z8, 0Y6N0Z9, 0Y6N0ZB, 0Y6N0ZC, 0Y6N0ZD, 0Y6N0ZF, 0Y6H0Z3, 0Y6J0Z3, 0Y6F0ZZ, 0Y6G0ZZ, 0Y670ZZ, 0Y680ZZ, 0Y620ZZ, 0Y630ZZ, 0Y640ZZ  Excluded:S78.xx, S88.xx, S98.xx |

# **Appendix 1. Construction of Age-Standardized Rate**

The methods used rates for this study draws on previous work using similar data and methods. ^4, 5^

We construct age-adjusted rate ratios in the following steps:

1. Calculate year, state, and subpopulation specific Medicaid enrollee counts using the American Community Survey (ACS)
2. Calculate Crude ED and IP rates for each year, state and subpopulation
3. Calculate Standardize rates, standard errors and 95% Confidence Intervals
4. Rate Ratios

Calculate Crude ED and IP rates

Following Newman ^6^ the crude ED utilization rate using SEDD data

𝑅=10,000∙𝑆𝑁

Where *S* is the number of estimated counts, and *N* is the corresponding (constant) denominator population.

The standard error of rates was calculated as:

𝑆𝐸𝑅=10,000 𝑆𝐸𝑆/𝑁

Note: Assume Poisson distribution of counts for variance estimation.

Standardization of Rates using the Direct Method

*Standard Population* 2010 CDC National Mortality Database.

Following Newman ^6^

𝑅𝑠𝑡𝑑=𝑆𝑡𝑎𝑛𝑑𝑎𝑟𝑑𝑖𝑧𝑒𝑑 𝑅𝑎𝑡𝑒= Σ𝑤𝑖𝑃𝑖𝑖

𝑣𝑎𝑟𝑠𝑡𝑑=𝑉𝑎𝑟𝑖𝑎𝑛𝑐𝑒 (𝑆𝑡𝑎𝑛𝑑𝑎𝑟𝑑𝑖𝑧𝑒𝑑 𝑅𝑎𝑡𝑒)= Σ𝑤𝑖2∗𝑉𝑎𝑟𝑖𝑎𝑛𝑐𝑒 (𝑃𝑖)𝑖

95% confidence intervals: (𝑅𝑠𝑡𝑑−1.96∗√𝑣𝑎𝑟𝑠𝑡 , 𝑅𝑠𝑡𝑑+1.96∗√𝑣𝑎𝑟𝑠𝑡𝑑)

Where *i* = 1 to 5 standard population bins, *wi* is the proportion of the standard population in population group *i* of the total standard population*,* and *Pi* is the estimated age-specific crude rate.

Rate Ratios

Following Newman ^6^ rate ratios to compare rates of ED/IP, use between race/ethnic subgroups.

𝑅𝑅= 𝑅1 /𝑅2

𝑉𝑎[log(𝑅𝑅)]= 𝑉𝑎𝑟(𝑅1)/(𝑅1)2+𝑉𝑎𝑟(𝑅2)/(𝑅2)2

95% confidence intervals: (exp(log(𝑅𝑅)−1.96∗√𝑉𝑎𝑟[log(𝑅𝑅)] ), exp(log(𝑅𝑅)+1.96∗√𝑉𝑎𝑟[log(𝑅𝑅)]))

Where 𝑅1 is the rate being compared and 𝑅2 is the reference rate.

# **eTable 2 Age-adjusted Rates of Diabetes Specific ED Visits per 10,000 Adult Medicaid Enrollees by Race/Ethnicity**

# **eTable 3 Age-adjusted Rates of Diabetes-Specific IP Hospitalizations Visits per 10,000 Adult Medicaid Enrollees by Race/Ethnicity**

# **eTable 4 Most Common Diagnostic Codes by Race/Ethnicity: ED**

| **Race/Ethnicity** | **Primary Diagnosis Code^a^** | **CPT Code** | **Frequency Rank** | **year** |
| --- | --- | --- | --- | --- |
| Black, Non-Hispanic | 25080 | 1371 | 1 | 2008 |
| Black, Non-Hispanic | 25002 | 611 | 2 | 2008 |
| Black, Non-Hispanic | 25060 | 385 | 3 | 2008 |
| Black, Non-Hispanic | 6826 | 281 | 4 | 2008 |
| Black, Non-Hispanic | 25010 | 136 | 5 | 2008 |
| Black, Non-Hispanic | 25080 | 2308 | 1 | 2011 |
| Black, Non-Hispanic | 25002 | 1188 | 2 | 2011 |
| Black, Non-Hispanic | 25060 | 772 | 3 | 2011 |
| Black, Non-Hispanic | 6826 | 595 | 4 | 2011 |
| Black, Non-Hispanic | 25081 | 265 | 5 | 2011 |
| Black, Non-Hispanic | 25080 | 2268 | 1 | 2014 |
| Black, Non-Hispanic | 25002 | 1352 | 2 | 2014 |
| Black, Non-Hispanic | 25060 | 1129 | 3 | 2014 |
| Black, Non-Hispanic | 6826 | 689 | 4 | 2014 |
| Black, Non-Hispanic | 25081 | 303 | 5 | 2014 |
| Hispanic, any race | 25080 | 435 | 1 | 2008 |
| Hispanic, any race | 25002 | 265 | 2 | 2008 |
| Hispanic, any race | 6826 | 138 | 3 | 2008 |
| Hispanic, any race | 25060 | 110 | 4 | 2008 |
| Hispanic, any race | 6827 | 59 | 5 | 2008 |
| Hispanic, any race | 25080 | 776 | 1 | 2011 |
| Hispanic, any race | 25002 | 514 | 2 | 2011 |
| Hispanic, any race | 6826 | 309 | 3 | 2011 |
| Hispanic, any race | 25060 | 259 | 4 | 2011 |
| Hispanic, any race | 25092 | 114 | 5 | 2011 |
| Hispanic, any race | 25080 | 977 | 1 | 2014 |
| Hispanic, any race | 25002 | 695 | 2 | 2014 |
| Hispanic, any race | 6826 | 497 | 3 | 2014 |
| Hispanic, any race | 25060 | 370 | 4 | 2014 |
| Hispanic, any race | 6827 | 164 | 5 | 2014 |

| White, Non-Hispanic | 25080 | 1820 | 1 | 2008 |
| --- | --- | --- | --- | --- |
| White, Non-Hispanic | 6826 | 732 | 2 | 2008 |
| White, Non-Hispanic | 25002 | 536 | 3 | 2008 |
| White, Non-Hispanic | 25060 | 396 | 4 | 2008 |
| White, Non-Hispanic | 25081 | 289 | 5 | 2008 |
| White, Non-Hispanic | 25080 | 2423 | 1 | 2011 |
| White, Non-Hispanic | 6826 | 1317 | 2 | 2011 |
| White, Non-Hispanic | 25002 | 871 | 3 | 2011 |
| White, Non-Hispanic | 25060 | 638 | 4 | 2011 |
| White, Non-Hispanic | 6827 | 406 | 5 | 2011 |
| White, Non-Hispanic | 25080 | 2711 | 1 | 2014 |
| White, Non-Hispanic | 6826 | 1739 | 2 | 2014 |
| White, Non-Hispanic | 25002 | 1147 | 3 | 2014 |
| White, Non-Hispanic | 25060 | 914 | 4 | 2014 |
| White, Non-Hispanic | 25013 | 592 | 5 | 2014 |
| Source: Author analysis of HCUP SEDD databases for non-elderly adults insured with Medicaid coverage, a diabetes diagnosis and a potentially preventable diabetes-specific condition. ^a^ ICD-9 Code | | | | |

| **Race/Ethnicity** | **Primary Diagnosis Code^a^** | **CPT Code** | **Frequency Rank** | **year** |
| --- | --- | --- | --- | --- |

| **Race/Ethnicity** | **Primary Diagnosis Code^b^** | **CPT Code** | **Frequency Rank** | **Data Year** |
| --- | --- | --- | --- | --- |
| Black, Non-Hispanic | E1165 | 9907 | 1 | 2017 |
| Black, Non-Hispanic | E11649 | 1783 | 2 | 2017 |
| Black, Non-Hispanic | E1065 | 1249 | 3 | 2017 |
| Black, Non-Hispanic | E1140 | 555 | 4 | 2017 |
| Black, Non-Hispanic | E11621 | 508 | 5 | 2017 |
| Hispanic, any race | E1165 | 4882 | 1 | 2017 |
| Hispanic, any race | E11649 | 636 | 2 | 2017 |
| Hispanic, any race | E1065 | 562 | 3 | 2017 |
| Hispanic, any race | E11621 | 332 | 4 | 2017 |
| Hispanic, any race | E10649 | 232 | 5 | 2017 |
| White, Non-Hispanic | E1165 | 9661 | 1 | 2017 |
| White, Non-Hispanic | E1065 | 2240 | 2 | 2017 |
| White, Non-Hispanic | E11649 | 1830 | 3 | 2017 |
| White, Non-Hispanic | E11621 | 871 | 4 | 2017 |
| White, Non-Hispanic | E1010 | 867 | 5 | 2017 |
| Source: Author analysis of HCUP SEDD databases for non-elderly adults insured with Medicaid coverage, a diabetes diagnosis and a potentially preventable diabetes-specific condition. ^b^ ICD-10 Code | | | | |

**eTable 5 Most Common Diagnostic Codes by Race/Ethnicity: IP**

| **Race/Ethnicity** | **Primary Diagnosis Code^a^** | **CPT Code** | **Frequency Rank** | **year** |
| --- | --- | --- | --- | --- |
| Black, Non-Hispanic | 25013 | 1455 | 1 | 2008 |
| Black, Non-Hispanic | 25002 | 1183 | 2 | 2008 |
| Black, Non-Hispanic | 25080 | 1003 | 3 | 2008 |
| Black, Non-Hispanic | 25012 | 881 | 4 | 2008 |
| Black, Non-Hispanic | 25082 | 564 | 5 | 2008 |
| Black, Non-Hispanic | 25013 | 2208 | 1 | 2011 |
| Black, Non-Hispanic | 25080 | 1198 | 2 | 2011 |
| Black, Non-Hispanic | 25002 | 1063 | 3 | 2011 |
| Black, Non-Hispanic | 25012 | 1029 | 4 | 2011 |
| Black, Non-Hispanic | 25062 | 770 | 5 | 2011 |
| Black, Non-Hispanic | 25013 | 2899 | 1 | 2014 |
| Black, Non-Hispanic | 25012 | 1292 | 2 | 2014 |
| Black, Non-Hispanic | 25080 | 1031 | 3 | 2014 |
| Black, Non-Hispanic | 25082 | 900 | 4 | 2014 |
| Black, Non-Hispanic | 25002 | 885 | 5 | 2014 |
| Hispanic, any race | 25002 | 753 | 1 | 2008 |
| Hispanic, any race | 25013 | 644 | 2 | 2008 |
| Hispanic, any race | 6826 | 490 | 3 | 2008 |
| Hispanic, any race | 25080 | 463 | 4 | 2008 |
| Hispanic, any race | 25012 | 350 | 5 | 2008 |
| Hispanic, any race | 25013 | 985 | 1 | 2011 |
| Hispanic, any race | 25002 | 611 | 2 | 2011 |
| Hispanic, any race | 25080 | 608 | 3 | 2011 |
| Hispanic, any race | 6826 | 518 | 4 | 2011 |
| Hispanic, any race | 25082 | 470 | 5 | 2011 |
| Hispanic, any race | 25013 | 1333 | 1 | 2014 |
| Hispanic, any race | 25080 | 665 | 2 | 2014 |
| Hispanic, any race | 25082 | 659 | 3 | 2014 |
| Hispanic, any race | 25012 | 548 | 4 | 2014 |
| Hispanic, any race | 6826 | 537 | 5 | 2014 |

#

#

| **Race/Ethnicity** | **Primary Diagnosis Code^b^** | **CPT Code** | **Frequency Rank** | **Data Year** |
| --- | --- | --- | --- | --- |

|  |  |  |  |  |
| --- | --- | --- | --- | --- |
| White, Non-Hispanic | 25080 | 1820 | 1 | 2008 |
| White, Non-Hispanic | 6826 | 732 | 2 | 2008 |
| White, Non-Hispanic | 25002 | 536 | 3 | 2008 |
| White, Non-Hispanic | 25060 | 396 | 4 | 2008 |
| White, Non-Hispanic | 25081 | 289 | 5 | 2008 |
| White, Non-Hispanic | 25080 | 2423 | 1 | 2011 |
| White, Non-Hispanic | 6826 | 1317 | 2 | 2011 |
| White, Non-Hispanic | 25002 | 871 | 3 | 2011 |
| White, Non-Hispanic | 25060 | 638 | 4 | 2011 |
| White, Non-Hispanic | 6827 | 406 | 5 | 2011 |
| White, Non-Hispanic | 25080 | 2711 | 1 | 2014 |
| White, Non-Hispanic | 6826 | 1739 | 2 | 2014 |
| White, Non-Hispanic | 25002 | 1147 | 3 | 2014 |
| White, Non-Hispanic | 25060 | 914 | 4 | 2014 |
| White, Non-Hispanic | 25013 | 592 | 5 | 2014 |
| Source: Author analysis of HCUP SEDD databases for non-elderly adults insured with Medicaid coverage, a diabetes diagnosis and a potentially preventable diabetes-specific condition. ^a^ ICD-9 Code | | | | |

| **Race/Ethnicity** | **Primary Diagnosis Code^b^** | **CPT Code** | **Frequency Rank** | **Data Year** |
| --- | --- | --- | --- | --- |
| Black, Non-Hispanic | E1010 | 1210 | 1 | 2017 |
| Black, Non-Hispanic | E1310 | 594 | 2 | 2017 |
| Black, Non-Hispanic | E1169 | 308 | 3 | 2017 |
| Black, Non-Hispanic | E11621 | 209 | 4 | 2017 |
| Black, Non-Hispanic | E1165 | 209 | 4 | 2017 |
| Hispanic, any race | E1010 | 4334 | 1 | 2017 |
| Hispanic, any race | E1310 | 2112 | 2 | 2017 |
| Hispanic, any race | E1169 | 2068 | 3 | 2017 |
| Hispanic, any race | E11621 | 1298 | 4 | 2017 |
| Hispanic, any race | E1165 | 759 | 5 | 2017 |
| White, Non-Hispanic | E1010 | 8283 | 1 | 2017 |
| White, Non-Hispanic | E1169 | 2497 | 2 | 2017 |
| White, Non-Hispanic | E1310 | 2288 | 3 | 2017 |
| White, Non-Hispanic | E11621 | 1562 | 4 | 2017 |
| White, Non-Hispanic | E1165 | 814 | 5 | 2017 |
| Source: Author analysis of HCUP SID databases for non-elderly adults insured with Medicaid coverage, a diabetes diagnosis and a potentially preventable diabetes-specific condition. ^b^ ICD-10 Code | | | | |

# **eTable 6 Age-adjusted Rates of All-Cause ED Visits per 10,000 Adult Medicaid Enrollees by Race/Ethnicity**

# **eTable 7 Age-adjusted Rates of All-Cause IP Hospitalizations Visits per 10,000 Adult Medicaid Enrollees by Race/Ethnicity**

# **eFigure 1 State All-Cause ED Visits for Adult Medicaid Enrollees with Diabetes by Race/Ethnicity**

**
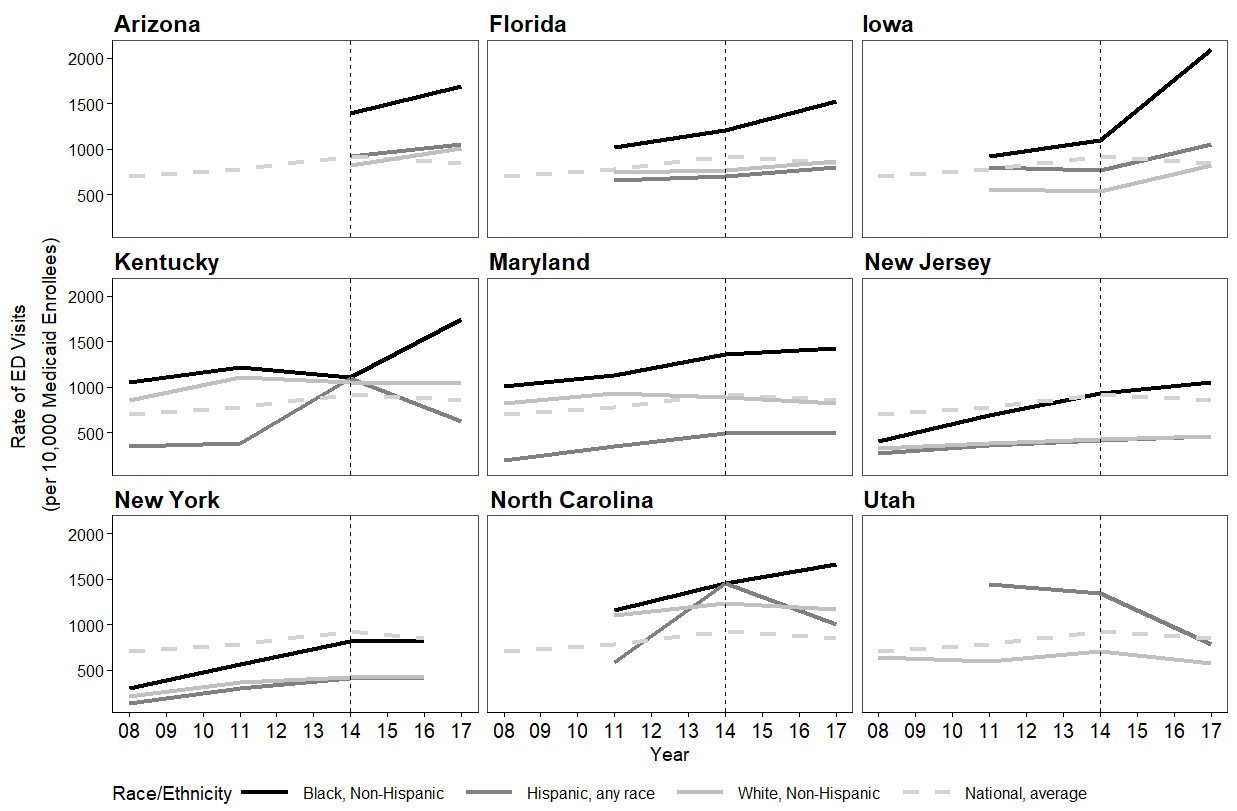
**

Source/Notes: Source: Author’s analysis of ED visits from SEDD and NED HCUP databases for the years: 2008, 2011, 2014 and 2017. Notes: States included in averages are Arizona, Utah, Nebraska, Iowa, Kentucky, North Carolina, Maryland, New Jersey, New York, Vermont, and Maryland. Rates calculated using state-year, race-specific age-adjusted population estimates derived from the ACS. Only individuals with a diabetes diagnosis are included in the sample. NEDS data was used to estimate national diabetes-related ED visits for all non-elderly adult Medicaid enrollees.

**eFigure 2 State All-Cause IP Visits for Adult Medicaid Enrollees with Diabetes by Race/Ethnicity**


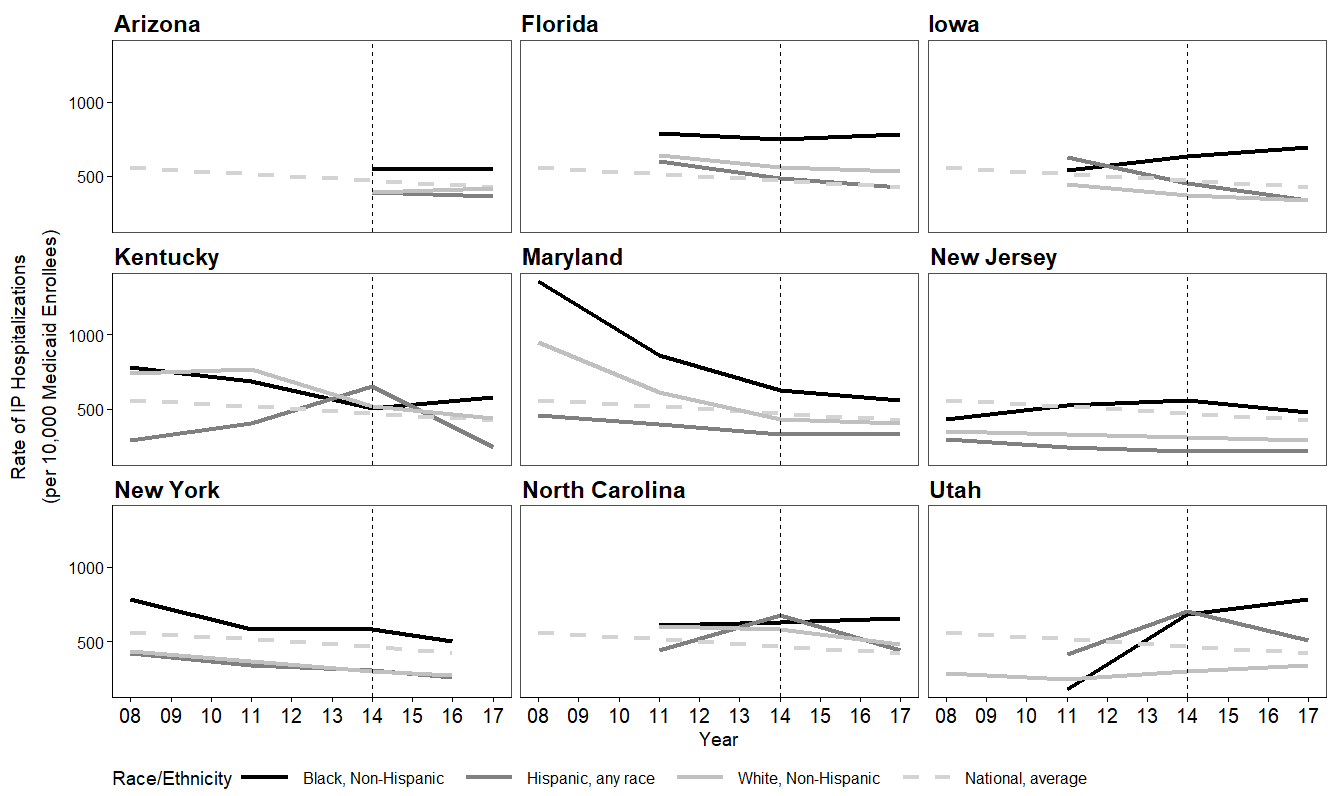


Source/Notes: Source: Author’s analysis of ED visits from SEDD HCUP databases and IP hospitalizations from SID HCUP databases for the years: 2008, 2011, 2014 and 2017. Notes: States included in averages are Arizona, Utah, Nebraska, Iowa, Kentucky, North Carolina, Maryland, New Jersey, New York, Vermont, and Maryland. Rates calculated using state-year, race-specific age-adjusted population estimates derived from the ACS. Only individuals with a diabetes diagnosis are included in the sample.

**eTable 8 Rate Ratios of Average Annual Recorded Emergency Department Visits - All Cause**

**eTable 9 Rate Ratios of Average Annual Recorded Inpatient Hospitalizations - All Cause**

1. Agency For Healthcare Research and Quality (AHRQ). Prevention Quality Indicators. Accessed 2020, January. https://www.qualityindicators.ahrq.gov/Modules/PQI_TechSpec_ICD10_v2020.aspx

2. Tseng C-L, Soroka O, Pogach LM. An expanded prevention quality diabetes composite: Quantifying the burden of preventable hospitalizations for older adults with diabetes. *Journal of Diabetes and its Complications*. 2018/05// 2018;32(5):458-464. doi:10.1016/j.jdiacomp.2018.01.013

3. Harding JL, Andes LJ, Rolka DB, et al. National and State-Level Trends in Nontraumatic Lower-Extremity Amputation Among U.S. Medicare Beneficiaries With Diabetes, 2000-2017. *Diabetes Care*. Oct 2020;43(10):2453-2459. doi:10.2337/dc20-0586

4. Uppal T, Chehal PK, Fernandes G, et al. National Trends in Emergency Department Use related to Diabetes, and Variation Across States and by Sociodemographic Factors, 2008-2017. *Fourthcoming JAMA Network Open*. 2022;

5. Turbow SD, Uppal TS, Haw JS, et al. Trends and Demographic Disparities in Diabetes Hospital Admissions: Analyses of Serial Cross-Sectional National and State Data, 2008-2017. *Diabetes Care*. Apr 5 2022;doi:10.2337/dc21-1837

6. Newman SC. *Biostatistical methods in epidemiology*. John Wiley & Sons; 2003.
